# Engaging patients, enhancing patient experiences: insights, innovations, and applications

**DOI:** 10.1186/s12913-018-3675-8

**Published:** 2018-12-14

**Authors:** Stephen J. O’Connor, Katherine A. Meese

**Affiliations:** Department of Health Services Administration, University of Alabama at Birmingham, School of Health Professions Building, 5th Floor, 1720 2nd Avenue South, Birmingham, AL 35294 USA

Though marketing is now a prevalent practice in the healthcare industry, this supplement of BMC Health Services Research by authors James K. Elrod and John L. Fortenberry, Jr. takes us back to a time when marketing within healthcare was neither industry standard nor frequently utilized. Furthermore, many in the industry viewed it as taboo. They describe the innovative process by which the Willis-Knighton Health System of Shreveport, Louisiana looked beyond the walls of the healthcare industry and became early adopters of different marketing mechanisms to transform the hospital experience, attract new patients, increase market share, and gain market dominance. Many of these marketing mechanisms have been adopted nationwide and are now considered industry standard.

The first article, “Formulating productive marketing communications strategy: a major health system’s experience,” details the process by which Willis-Knighton Health System bucked the industry norm by aggressively pursuing a marketing, communications, and advertising strategy. They creatively utilized billboards to communicate important offerings and health information, such as immunization programs available in the community. Instead of relying on press outlets to craft the story for the institution, Willis-Knighton Health System took matters into their own hands to communicate directly with the consumer. This strategy, uncommon in healthcare at the time, is credited by Willis-Knighton Health System as a key driver for engaging patients and gaining market dominance.

The next article, “Establishing Good Samaritan programs in healthcare institutions: a method for enhancing patient experiences and increasing loyalty,” describes a unique concierge-type program designed to both improve patient loyalty to Willis-Knighton Health System and also address social determinants of health by reducing barriers to accessing care. Willis-Knighton Health System’s Good Samaritan program, named after the biblical story which epitomizes care and compassion towards others, goes to extraordinary lengths to address the needs, wants, and comfort of patients. This program not only highlights creative approaches to serving patients, but also represents an example of a marketing and customer loyalty strategy that demonstrates compassion and care for all challenges a patient may face on the road to health.

“Target marketing in the health services industry: the value of journeying off the beaten path” describes an innovative approach to marketing, and the importance of creatively marketing to different consumer segments. Specifically, the article profiles Willis-Knighton Health System’s Pediatric Orientation Program, an educational offering for children which familiarizes them to the hospital and provides various insights on healthy living. The program also introduces them to possible careers in the healthcare field. The Pediatric Orientation Program’s aim, in addition to being educational, is to reduce children’s fear associated with a hospital visit and to foster loyalty among children and parents. This program serves as an excellent example of a marketing innovation benefiting both the patient and the hospital.

“Driving brand equity in health services organizations: the need for an expanded view of branding” recounts the development of a unique branding approach designed for maternal and child health services. After inspiration from the retail industry, Willis-Knighton Health System developed a unique patient gift—Willis the Bear, a branded stuffed animal—to both commemorate the birth of a child and serve as an enduring keepsake. The item’s popularity gained recognition within the region and essentially became a product placement mechanism within the mother and child’s home. Willis-Knighton Health System credits this innovation with helping secure and grow labor and delivery patient volumes.

“Healthcare establishments as owner-operators of digital billboards: making the most of excellent roadside visibility and high traffic counts to better connect with patients” details the process of purchasing and utilizing digital billboards as a patient engagement mechanism. In addition to the brand-promoting billboards described in the introduction, Willis-Knighton Health System uses its digital billboards to communicate important patient information like vaccine schedules, community health fairs, and wellness tips. This patient-focused approach represents an innovative way of using a traditional marketing mechanism to engage and inform patients with the aim of improving the health of the community. While the brand is promoted, the way Willis-Knighton Health System deploys billboards goes beyond pure advertising to provide beneficial and useful information to patients.

In “Am I seeing things through the eyes of patients? An exercise in bolstering patient attentiveness and empathy,” readers get a glimpse into the Willis-Knighton Health System approach to helping all staff members orient themselves towards the patient. This is one of a range of tools used by Willis-Knighton Health System to encourage employees to deliver their very best. Over time, these tools have helped embed patient-centered mindsets into the culture and practices of the institution.

Lastly, “Catalyzing marketing innovation and competitive advantage in the healthcare industry: the value of thinking like an outsider” summarizes Willis-Knighton Health System’s various innovations to explain how creative thinking can reap myriad benefits including competitive advantages. Beyond specific marketing and communications tools, Willis-Knighton Health System, driven by creative experimentation, highly values looking outside of the healthcare industry for innovations and insights that can be used within.

It is important to note, as with any case study research, that the specific innovations used by Willis-Knighton Health System may not be the optimal strategic choice for health systems elsewhere. Specific market dynamics, patient demographics, and preferences may warrant different approaches. Similarly, transportation patterns, local competition, and hospital capabilities should all be considered when developing a marketing strategy. Additionally, some countries heavily regulate whether doctors or hospitals can advertise at all. However, the overarching theme of Willis-Knighton Health System’s approach, which is indeed universally applicable, is the importance of a willingness to look outside of the industry, experiment, take risks, and learn as an organization. The unifying theme through each article about Willis-Knighton Health System is that the institution’s leadership both valued and encouraged experimentation, affording new ideas in marketing and communications. They drew heavily on experiences from the retail and hotel industry and took an aggressive approach to marketing at a time when it was uncommon in the healthcare industry. Willis-Knighton Health System credits this bold experimentation to significantly improving patient engagement, experience, and volumes, ultimately giving the institution a dominant position in the region.

